# Simpson−Golabi−Behmel syndrome type 1 with subclinical hypothyroidism: A case report

**DOI:** 10.1097/MD.0000000000017616

**Published:** 2019-10-25

**Authors:** Jing Zhang, Kai Mu, Haiyan Xu, Yuehua Guo, Zhijie Liu, Liling Wang, Jiahui Li, Fengjuan Zhang, Yan Kou, Xin Yuan

**Affiliations:** a Department of Pediatrics, Shandong Provincial Qianfoshan Hospital, The First Hospital Affiliated with Shandong First Medical University; b Department of Pediatrics, Shandong Provincial Qianfoshan Hospital, Shandong University, Jinan, P.R. China.

**Keywords:** gPC3, Simpson−Golabi−Behmel syndrome type1, subclinical hypothyroidism

## Abstract

**Rationale:**

Simpson−Golabi−Behmel syndrome type 1 (SGBS1) is caused by mutations in GPC3 or in both GPC3 and GPC4. Physical manifestations of SGBS1 include fetal overgrowth and macrostomia, macroglossia. Subclinical hypothyroidism has never been reported in SGBS1 cases.

**Patient concerns:**

An 8-days-old boy was referred to our hospital with persistent hypoglycemia and special facies. And the infant showed elevated levels of thyroid-stimulating hormone (TSH). Free T4 and free T3 were normal.

**Diagnoses:**

Definitive diagnosis of SGBS1 depends on clinical features and genetic testing. A nonsense mutation (c.1515C > A, p. Cys505∗) was tested by whole-exome sequencing.

**Interventions:**

Normal blood glucose levels were maintained with glucose infusions. Levothyroxine was given to the patient for treating subclinical hypothyroidism.

**Outcomes:**

The parents decided to abandon the treatment of the patient. We learned that the patient died of a lung infection by a telephone follow-up.

**Lessons:**

Subclinical hypothyroidism could be added to the known clinical manifestations of SGBS1.

## 1 Introduction

First reported by Simpson et al in 1975,^[1]^ Simpson−Golabi−Behmel syndrome type 1 (SGBS1, OMIM#312870) is caused by mutations in the GPC3 or in both GPC3 and GPC4.^[2]^ SGBS1 is characterized by a range of clinical manifestations including macrosomia, distinctive facies (including coarse facial features, macrostomia, and macroglossia), and polydactyly.^[3,4]^ Diagnosis of SGBS1 in males is established by observable clinical manifestations and/or detection of a pathogenic variant of GPC3, or a large duplication of GPC3 and/or GPC4.^[5,6]^ In the present report, we describe a neonate of SGBS1 with a nonsense mutation in GPC3 presenting hypothyroidism and subclinical hypothyroidism.

## 2 Case report

Informed consent was obtained from the patient’ parents for the publication of this case report and its accompanying images. The male patient was referred to our hospital at the age of 8 days presenting hypoglycemia and special facies. The patient had been delivered vaginally at 37 + 2/7 weeks’ gestational age with a birth weight of 4200 g (>95th centile). His birth length was 53 cm (75th–90th centile), and occipital frontal head circumference of 34.5 cm (50th–90th centile). The patient's Apgar score was determined at 1, 5, and 10 minutes and rated 10 each time. The patient's mother was 32 years old and undergone regular pregnancy checks. The patient's father age was 33 years old. Both parents were healthy and there was no family history of genetic disease.

**Figure 1:**
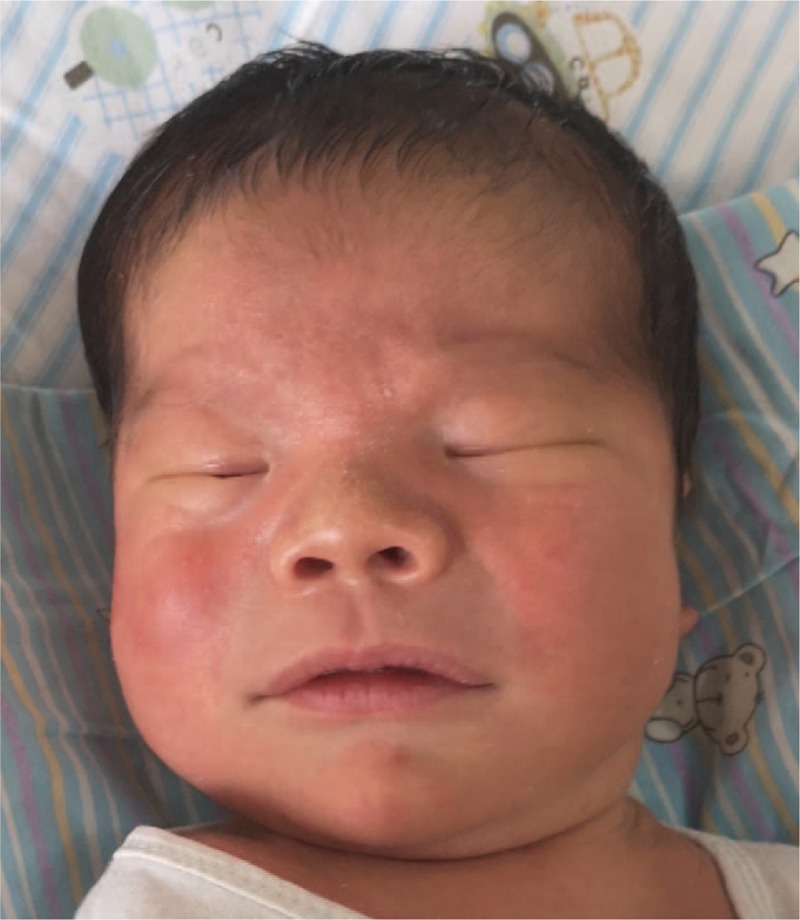
The patient exhibited macrocephaly, macrostomia, and coarse facial features.

The patient had persistent hypoglycemia after birth with normal enteral nutrition. Normal blood glucose levels were maintained with glucose infusions. The infant was transferred to our hospital to ascertain the cause of hypoglycemia and distinctive craniofacial features. Physical examination revealed coarse skin, and desquamate could be seen in the patient's wrinkles. The trunk and limbs had scattered red maculopapular rashes. The patient also exhibited macrostomia (Fig. 1), macroglossia, and hepatomegaly. The liver was 2 and 1 cm below the right costal margin and the xiphoid, respectively, and had a soft texture, as determined by palpation. The spleen was not palpated below the left costal margin. The anus opening was normal and hypertrophy was noted in the perianal fold. The fingers were thick, and the patient had postaxial polydactyly.

Normal results were obtained from laboratory examinations, including fecal examination, as well as routine blood, liver function, renal function, serum insulin, and C peptide tests. Routine urine testing showed positive (1+) urinary protein and elevated urinary microalbumin. A high level of thyroid-stimulating hormone (TSH) was found at the age of 14 days, showing that serum TSH was 9.650 μ/L (normal range 0.27–4.2 μ/L). Free T4 and free T3 were normal. No special treatments for high level of TSH were given to the patient. Antithyroid peroxidase autoantibody and antithyroglobulin antibodies were negative. The thyroid function test was repeated after 5 days, and serum TSH was higher than in the initial test (TSH 27.11 μ/L). Free T3 was 2.88 μ/L (normal range 3–8.1 μ/L) and free T4 was 14.19 μ/L (normal range 12–22 μ/L). Levothyroxine was given to the patient. Echocardiography showed normal diameter of each compartment. Abdominal ultrasound showed hepatomegaly and hydrocele. We consider that the patient may have either Beckwith−Wiedemann syndrome^[7]^ or methylmalonic acidemia and performed blood tandem mass spectrometry and whole exon sequencing for verification. The result of blood tandem mass spectrometry was normal, but whole exome sequencing revealed a hemizygote genetic mutation in GPC3 (chrX: 132730526, NM_004484.3: c.1515C > A, p. Cys505∗). The mutation was verified by Sanger sequencing. Neither parent was found to have the mutation, so the mutation found in the infant was de novo. The parents did not want to continue the treatment for the infant. Parents signed the consent of abandoning treatment and requested discharge for the patient. We learned that the patient died of a lung infection by a telephone follow-up.

## 3 Discussion

The case of SGBS1 has not been reported in China. The patient's condition was consistent with typical clinical manifestations of SGBS1, such as postnatal macrosomia, coarse facial features, macrostomia, macroglossia, and polydactyly.^[8]^ The clinical phenotype also included subclinical hypothyroidism, which has not been reported in other case reports or literature related to SGBS1. Subclinical hypothyroidism refers to that serum TSH is above the upper limit of normal range, while free T4 is normal.^[9]^ A retrospective study including 1416 children and adults showed that TSH levels were positively correlated with insulin.^[10]^ However, some pediatric studies have shown that subclinical hypothyroidism is not associated with insulin sensitivity.^[11,12]^ The mechanism by which SGBS1 causes this subclinical hypothyroidism remains unclear and warrants further investigation.

Furthermore, the patient had persistent hypoglycemia even when provided enteral nutrition in accordance with his age and weight. Although hypoglycemia is a known clinical characteristic of neonatal SGBS1 in the review,^[3]^ detailed clinical case reports have not been searched. Experimental studies have shown that adipocytes of SGBS patients are more sensitive to insulin stimulation, which may increase glucose uptake and cause hypoglycemia.^[13]^ GPC3, a member of the glypican family, regulates hedgehog signaling of fibroblast growth factors.^[14]^ GPC3 is expressed in a tissue-specific manner, exhibiting peak expression during embryonic tissue development and downregulated in mature tissues.^[15]^ As glypicans are expressed predominantly during development, they are thought to play a role in morphogenesis.^[16]^ The patient in the current study harbored a de novo mutation in GPC3 (c.1515C > A, p. Cys505∗), which resulted in SGBS1. There was known disease mutation at this position (HGMD CD994337, c.1515del) in Japan.^[17]^

During a follow-up interview with the patient's family we learned that the child had died from a lung infection after being discharged from the hospital. GPC3 encodes a cell surface proteoglycan that is linked to the outer leaflet of the plasma membrane by a glycosyl phosphatidyl inositol.^[18,19]^ Proteoglycans in the extracellular matrix can interact with receptors on the surface of immune cells to form a defensive barrier against the harmful macromolecules.^[20,21]^ It seems plausible that the patient's susceptibility to infection would have been increased by his SGBS1, although we cannot be certain.

As above, subclinical hypothyroidism has never been reported in SGBS1 cases, and no other pathogenic mutation was found by WES which may cause subclinical hypothyroidism, the phenotype may be one of the clinical manifestations of SGBS1. Special types of mutations, such as complex recombination of genes and variations in the copy number of large fragments, cannot be excluded from the case in this study. In addition, DNA analyzed in the current study was peripheral blood and interpretation bias caused by chimerism cannot be excluded. Whether high TSH contributes to SGBS needs further investigation.

## Author contributions

**Conceptualization:** Haiyan Xu, Zhijie Liu, Fengjuan Zhang.

**Data curation:** Jing Zhang, Liling Wang, Fengjuan Zhang.

**Formal analysis:** Kai Mu.

**Project administration:** YueHua Guo, Jiahui Li.

**Resources:** Yan Kou.

**Writing – original draft:** Jing Zhang.

**Writing – review & editing:** Xin Yuan.
